# Effects of cow dung and wood biochars and green manure on soil fertility and tiger nut (*Cyperus esculentus* L.) performance on a savanna Alfisol

**DOI:** 10.1038/s41598-020-78194-5

**Published:** 2020-12-03

**Authors:** Aruna Olasekan Adekiya, Abiola Folakemi Olaniran, Titilayo Tolulope Adenusi, Charity Aremu, Wutem Sunny Ejue, Yetunde Mary Iranloye, Abiodun Gbadamosi, Adeniyi Olayanju

**Affiliations:** 1 College of Agricultural Sciences, Landmark University, PMB 1001, Omu-Aran, Kwara State Nigeria; 2 Department of Food Science and Nutrition, Landmark University, PMB 1001, Omu-Aran, Kwara State Nigeria; 3 Department of Agricultural and Bio-System Engineering, Landmark University, PMB 1001, Omu-Aran, Kwara State Nigeria

**Keywords:** Plant sciences, Environmental sciences

## Abstract

Two field experiments were conducted concurrently in 2019. The study investigated the effects of single and combined application of wood biochar (WB) and cow dung biochar (CDB) with green manure (GM) on soil properties, performance, and tuber qualities of tiger nut. The treatments consisted of: CDB at 10 t ha^−1^, WB at 10 t ha^−1^, GM—*Tithonia diversifolia* at 10 t ha^−1^, CDB + GM, WB + GM, control. The six treatments were arranged in RCBD with three replications. CDB, WB, and GM either sole or combined increased moisture content, SOC, nutrient contents, culturable microorganisms, performance, moisture, ash, fiber, and protein contents of the tiger nut compared with the control. CDB has a higher N, C: N ratio, P, K, Ca, Mg, CEC, and pH relative to. CDB alone and CDB + GM increased growth and yield compared with WB alone and WB + GM. CDB + GM has the highest value of growth and yield of tiger nut. GM alone improved growth and yield of tiger nut compared with the two sole biochar treatments. CDB + GM and WB + GM increased growth and yield of tiger nut compared with their sole forms. This was adduced to biochar allowing the retention of nutrients from rapidly decomposing *Tithonia* within the rooting zone, thereby promoting better effectiveness of nutrient uptake and increase in yield. Therefore, for good soil fertility and tiger nut yield, it is important that the addition of a fast releasing nutrient source to biochar be sought.

## Introduction

Tiger nut (*Cyperus esculentus* L.) is a tuber crop belonging to the family Cyperaceae. They produce edible tubers with a sweet flavor^1^. Tiger nut is unexploited owing to lack of information on their nutritive prospective. Nevertheless, the tuber is rich in dietary fiber, carbohydrate, protein, iron, calcium, and oil^2–4^. The oil of the tiger nut tuber contains high quantity of unsaturated fatty acids and therefore, has a superb nutritive potentials with a fat content comparable to that of olives^5^.

In Nigeria, tiger nut is mainly grown in the middle belt and northern region^6^. The soils of this region are mostly Alfisols, Inceptisols, and Ultisols, the latter especially characterized by low activity clays, low organic matter content, and high sand content, thus these soils are physically fragile and susceptible to degradation^7^. This low soil fertility status usually leads to very low crop yields on farmers’ fields. Experimental results have shown that these inherent poor soil fertility of these savanna soils can be overcome by management strategies^8^. One such strategy is the use of organic amendments.

Organic amendments, such as biochar and green manure, could be a useful strategy to sustainably maintain or increase soil organic matter content, preserve the physical nature of the soil and improve soil fertility and crop yield. Biochar is the product of the thermochemical conversion of organic materials with a small amount of oxygen at high, low, or intermediate temperatures^9^. Biochar is stable, rich in nutrients, and it can persist in soil for many years^10,11^. Biochar also provides a number of soil health benefits, such as; increased soil organic matter, improved soil structural stability, reducing nutrient leaching, provide greater nutrient availability in soil, and improve the efficiency of nutrient utilization in crops, increasing the amount and structural diversity of microbes in applied soils^12^.

Biochar can be produced from a wide range of biomass sources such as woody plant materials as well as agricultural wastes including manures. The most important pointer of biochar quality is its high adsorption and cation exchange capacities, pH and low levels of mobile matter and high aromatic carbon content^13–15^ and these qualities are more dependent on the feedstock characteristics.

Plant-derived biochars have high aromatic C content due to the greater amount of lignin and cellulose present, which gives the biochar high stability and resistance to microbial decomposition^16,17^. Animal manures have high contents of labile organic and inorganic compounds, resulting in biochars with high ash content, which is positively related to the nutrient and chemical composition of the biomass^18^. In Nigeria, research has hitherto been concentrated on the use of plant-derived biochar, there is the need to investigate the use of cow dung as biochar.

Green manures have been reported^19^ to improve soil fertility by its imprint on soil organic matter, increase in nutrients in the soil and making them available near the soil surface, reduce nutrient leaching especially N and minimize erosion.

Similarly, biochar has been reported to improve soil fertility, it may however not be good as the only nutrient supplier due to its poor nutrient composition and its slow rate of degradation^9^. Therefore, it is imperative to find a fast decomposing amendment source to be added to biochar. Recent researches has shown that addition of biochar to organic amendments could lead to improved soil properties and crop yield compared with biochar alone^20^. However, as biochar produced from different organic sources have different characteristics, their effect on an addition to manure may be different. This also needs investigation.

In this work, mexican sunflower (*Tithonia diversifolia* Asteraceae) was chosen as green manure source due to its wide distribution throughout the humid and sub-humid tropics in Asia and Africa^21^, its relatively high nutrient concentrations (N, P, and K) that are found in its biomass because of its ability to extract the relatively high amount of nutrients from the soil and its rapid decomposition^22,23^. Therefore the objectives of this study were to investigate the effects of single and combined application of wood and cow dung biochar with green manure on soil properties, growth, yield, and tuber qualities of tiger nut on a tropical savanna Alfisol.

## Results

### Soil properties prior experimentation and analysis of biochars and green manure

Tables 1 and 2 respectively showed the results of the soil of the sites before experimentation and the chemical analysis of biochars and green manure. The soils of the two sites were sandy loam in texture, acidic, moderate in bulk density and low ins oil organic matter and nutrients (N, K, Ca, and Mg) except P according to the critical level of 3.0% OM, 0.20% N, 10.0 mg kg^−1^ available P, 0.16–0.20 cmol kg^−1^ exchangeable K, 2.0 cmol kg^−1^ exchangeable Ca, and 0.40 cmol kg^−1^ exchangeable Mg recommended for crop production in ecological zones of Nigeria^24^. In the soils of both sites, the order of occurrence of soil microbes were: bacteria > actinomycetes > fungi. Analysis of cow dung and wood biochar and green manure (Table 2) showed that cow dung biochar has higher pH and CEC compared with wood biochar. Among the three soil amendments, cow dung biochar has the highest Na, Mg, P, and green manure has the highest of Ca, K, and N. whereas wood biochar was highest in organic C and C: N ration.

**Table 1:** Pre-plant soil properties.

Property	Site A	Site B
Sand (%)	68.9	68.7
Silt (%)	15.9	16.1
Clay (%)	15.2	15.2
Textural class	Sandy loam	Sandy loam
Bulk density (g cm^−3^)	1.46	1.41
Organic matter (%)	2.64	2.60
pH (water)	5.61	5.66
Total N (%)	0.17	0.17
Available P (mg kg^−1^)	10.6	10.1
Exchangeable K (cmol kg^−1^)	0.13	0.12
Exchangeable Ca (cmol kg^−1^)	1.60	1.58
Exchangeable Mg (cmol kg^−1^)	0.35	0.34
Bacteria × 10^6^ (CFU/g)	1.34	1.31
Actinomycetes × 10^5^ (CFU/g)	1.50	1.51
Fungi × 10^3^ (CFU/g)	2.39	2.40

**Table 2:** Chemical analysis of cow dung biochar, wood biochar and green manure.

Property	Cow dung biochar	Wood biochar	Green manure
pH (water)	8.31a	7.31b	ND
Organic C (%)	35.2b	56.7a	27.8c
Total N (%)	1.15b	0.89c	3.88a
C: N ratio	30.61b	63.71a	7.16c
Ash (%)	0.51a	0.48b	ND
P (%)	1.54a	0.72b	0.48c
K (%)	3.66b	1.41c	4.41a
Ca (%)	1.38b	1.24c	3.42a
Mg (%)	1.36a	0.41b	0.11c
Na (%)	1.88a	0.68b	0.14c
CEC (cmol kg^−1^)	5.7a	3.1b	1.58c

Values followed by similar letters under the same row are not significantly different at *p* = 0.05 according to Duncan’s multiple range test.

### Effects of cow dung and wood biochars and green manures on soil properties

The effects of cow dung and wood biochars and green manures on soil chemical and moisture content and biological properties are respectively presented in Tables 3 and 4. CBD, WB, GM, CDB + GM, and WB + GM increased pH, organic C, N, P, K, Ca, Mg, CEC, and moisture contents of the soil significantly relative to the control. Among sole applications of CBD, WB, and GM, CBD and WB produced statistically similar but higher values of soil pH organic C and moisture content compared with GM. CBD, WB, and GM also produced statistically similar values of CEC. However, GM alone produced higher values of N, K, and Ca compared with CBD and WB. The combined application of CDB + GM and WB + GM increased organic C, N, P, K, Mg, CEC, and moisture content of the soil relative to their sole forms. In almost all cases (values of K, Ca and CEC for CDB + GM and WB + GM were not significantly different) CDB + GM increased soil nutrients compared with WB + GM. Soil microbes (bacteria, fungi, and actinomycetes) were significantly more abundant in amended soils compared with the control (Table 3). The values of CDM and WB were statistically similar and significantly higher than GM. The combined application of CDB + GM and WB + GM also increased soil bacteria, fungi, and actinomycetes compared with their sole forms.

**Table 3:** Effects of cow dung and wood biochars and green manures on soil chemical properties and moisture content.

Treatment	pH (water)		OC (%)		N (%)		P (mg kg^−1^)		K (cmol kg^−1^)		Ca (cmol kg^−1^)		Mg (cmol kg^−1^)		CEC (cmol kg^−1^)		Moisture content (%)	
	Site A	Site B	Site A	Site B	Site A	Site B	Site A	Site B	Site A	Site B	Site A	Site B	Site A	Site B	Site A	Site B	Site A	Site B
C	5.51c	5.55c	0.93d	0.91d	0.16f.	0.15f.	9.5f.	9.3f.	0.12e	0.11e	1.55e	1.49e	0.30f.	0.28f.	4.81e	4.73e	10.6d	10.9d
CDB	6.64a	6.73a	1.94a	1.93a	0.19d	0.18d	17.2c	17.9c	0.26c	0.24c	1.88c	1.80c	0.72c	0.71c	5.41c	5.42c	13.6b	13.8b
WB	6.60a	6.69a	1.90a	1.90a	0.18e	0.17e	15.6d	15.8d	0.24d	0.21d	1.71d	1.68d	0.51d	0.48d	5.18d	5.15d	13.2b	13.6b
GM	6.10b	6.15b	1.40c	1.44c	0.21c	0.21c	13.4e	13.9c	0.36b	0.34b	2.10b	2.00b	0.46e	0.44c	5.19d	5.09d	12.1c	12.4c
CDB + GM	6.60a	6.71a	1.98a	1.93a	0.29a	0.28a	20.6a	21.4a	0.41a	0.40a	2.46a	2.41a	0.88a	0.87a	5.96a	5.94a	14.6a	14.7a
WB + GM	6.59a	6.64a	1.91a	1.90a	0.25b	0.24b	18.1b	19.3b	0.40a	0.39a	2.40a	2.38a	0.78b	0.77b	5.69b	5.59b	14.3a	14.4a

Values followed by similar letters under the same column are not significantly different at *p* = 0.05 according to Duncan’s multiple range test.

**Table 4:** Mean population of soil culturable microorganisms.

Treatment	Bacteria × 10^6^ (CFU/g)		Actinomycetes × 10^5^ (CFU/g)		Fungi × 10^3^ (CFU/g)	
	Site A	Site B	Site A	Site B	Site A	Site B
Control	1.36d	1.39d	1.54d	1.58d	1.60d	2.43d
Cow dung biochar	8.51b	8.66b	3.64b	3.71b	3.33b	3.43b
Wood biochar	8.11b	8.25b	3.61b	3.66b	3.31b	3.39b
Green manure	6.23c	6.42c	1.95c	1.98c	2.10c	2.94c
Cow dung biochar + Green manure	10.4a	10.85a	5.41a	5.68a	4.66a	4.86a
Wood biochar + Green manure	10.12a	10.56a	5.10b	5.59ab	4.61a	4.78a

Values followed by similar letters under the same column are not significantly different at *p* = 0.05 according to Duncan’s multiple range test.

### Effects of cow dung and wood biochars and green manures on growth and yield parameters of tiger nut

Table 5 shows the data on the response of growth and yield of tiger nut to cow dung and wood biochars and green manures. CDB, WB, GM, CDB + GM, and WB + GM significantly increased plant height, number of leaves, number of tubers, and weight of tubers per plant compared with the control. GM had significantly higher growth and yield parameters of tiger nut compared with CDB and WB. The order of growth and yield among the amendment applied alone was: GM > CDB > WB. CDB + GM and WB + GM significantly increased growth and yield of tiger nut compared with their sole treatments. Among all treatments, CDB + GM has the highest value. Using the mean of the two sites, CDB + GM increased tiger nut weight by 36.1 and 24.5% respectively compared with CDB and GM. Similarly, WB + GM increased tiger nut weight by 47.5 and 14.0% respectively compared with WB and GM.

**Table 5:** Effects of cow dung and wood biochars and green manures on growth and yield parameters of tiger nut.

Treatment	Plant height (cm)		Number of leaves/plant		Number of tubers/plant		Weight of tuber/plant (g)	
	Site A	Site B	Site A	Site B	Site A	Site B	Site A	Site B
Control	26.5f.	28.1f.	6.8f.	6.9f.	22f.	23f.	24.2f.	23.6f.
Cow dung biochar	45.1d	51.6d	8.1d	8.3d	31d	30d	34.7d	35.1d
Wood biochar	41.8e	46.1e	7.5e	7.6e	28e	27e	30.1e	29.6e
Green manure	496c	55.6c	8.8c	9.1c	35c	34c	39.3c	38.1c
Cow dung biochar + Green manure	66.1a	63.3a	11.6a	12.1a	44a	46a	46.4a	48.5a
Wood biochar + Green manure	60.5b	59.1b	9.8b	10.6b	40b	41b	43.6b	44.6b

Values followed by similar letters under the same column are not significantly different at *p* = 0.05 according to Duncan’s multiple range test.

### Effects of cow dung and wood biochars and green manures on proximate composition of tiger nut

**Figure 1:**
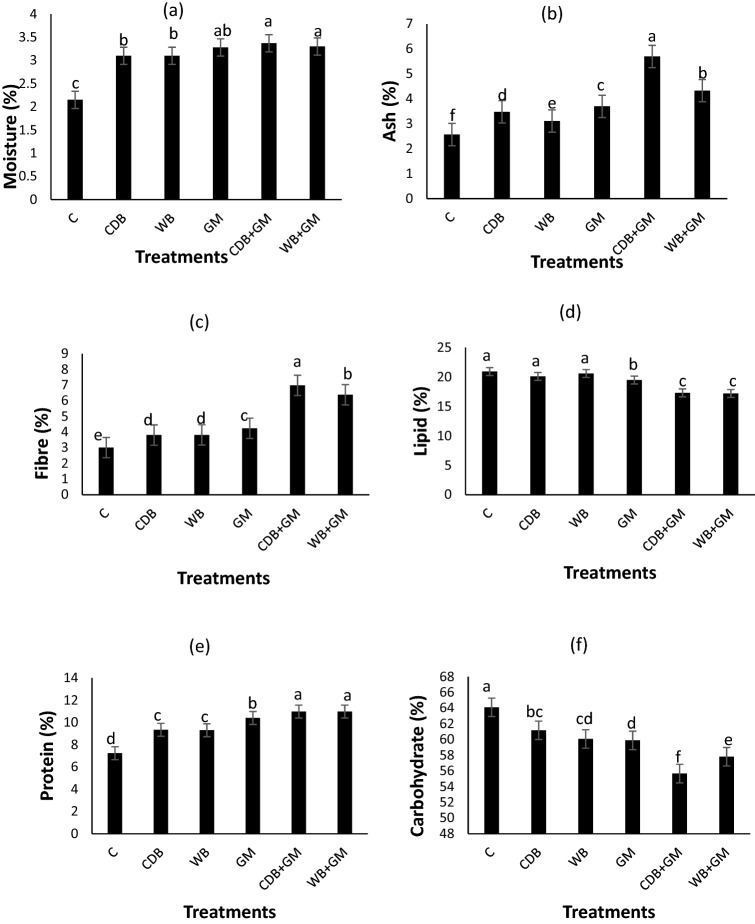
Effects of cow dung and wood biochars and green manures on proximate composition of tiger nut: *C* control; *CDB* cow dung biochar; *WB* wood biochar. Vertical bars show standard error of paired comparisons; bars marked with different letters show means significantly different at 5% level using Duncan's multiple range test.

The effects of cow dung and wood biochars and green manures on the proximate composition of tiger nut are presented in Fig. 1. Application of amendments either in sole or combined forms increased moisture, ash, fiber, and protein compared with the control. Application of amendments either in sole or combined forms reduced lipids and carbohydrates compared with the control. Except for lipid and carbohydrate, GM produced the highest values of proximate of tiger nut when compared with CDB and WB. Combinations of CDB + GM and WB + GM produced higher values of moisture, ash, fiber, and protein but reduced lipids and carbohydrates compared to their sole forms (CDB, WB, and GM). In all, CDB + GM produced the best values of moisture, ash, fiber, and protein.

## Discussion

The soils the sites prior to experimentation was low in nutrient. This state of the soil is the characteristic of Nigerian savanna soils. Salako^25^ reported that Nigeria savanna soils are low in organic matter and chemical fertility. The fairly high bulk density before the start of the experiment was partly due to its low organic matter^26^.

Sole application of CDB, WB, and GM and combinations CDB + GM and WB + GM increases the pH and nutrient contents of the soil. The enhancement of the soil chemical composition as a result of biochar was because biochar though inert contain some nutrients (Table 2) and again due to its high porosity and surface area is able to absorb soluble organic matter and inorganic nutrients^27^. The high retention capacity of biochar was due to the carboxylate groups present in biochar^28^. Biochar was found to be a good absorbent of soluble nutrients like ammonium, nitrate, phosphate, and other ionic solutes^29–32^. The cation exchange site present in biochar is responsible for its improvement in CEC compared with no biochar soils^33^, which could also be responsible to the retention of NH4^+^ and enhancement of N in biochar soils^34,35^. The pH of biochar treated plots increased because contain ash (Table 2) that are rich in K, Ca, Mg and Na which can raise pH^36^.

GM increased pH, OC, N, P, K, Ca, Mg, and CEC compared with the control (Table 3). This revealed that these manure degraded and nutrients in them are released to the soil. Shokalu et al.^37^ found that *Tithonia* significantly improved pH, N, P, K, Mg, and Zn contents of the soil.

The improved moisture content of the soil due to biochar was adduced to the porous nature of biochar which would have allowed it to retain water in its micro and mesopores^20^. Chan et al.^38^ also reported that the water retention ability of biochar could be as a result of an increase in overall net soil surface area in the soil after biochar application. GM improved moisture content due to improved soil aggregation that created pore spaces resulting from greater earthworm burrowing in the amended soil and hence improved moisture content.

Sole application of CDB, WB, and GM and combinations CDB + GM and WB + GM increased the abundance of soil microbes. This was as a result of the improvement in soil physical and chemical properties that control biological activities in the soil^39^, such as increased pH, moisture content, and retentions of major nutrients^14^. In fact for site A, the respective correlation between bacteria (r = 0.91, 0.75 & 0.89, *p* > 0.05), fungi (r = 0.87, 0.97 & 0.92, *p* > 0.05) and actinomycetes (r = 0.83, 0.94 & 0.95, *p* > 0.05) with soil pH, moisture content and CEC were positive. Likewise, for site B the respective correlation between bacteria (r = 0.94, 0.99 & 0.91, *p* > 0.05), fungi (r = 0.82, 0.95 & 0.93, *p* > 0.05) and actinomycetes (r = 0.33, 0.93 & 0.93, *p* > 0.05) with soil pH, moisture content and CEC were positive. It follows therefore that CDB and WB have more microbes compared to GM because of improved pH, moisture content, and CEC. Also, the porous nature and adsorption properties of biochar may provide a favorable environment for the growth and reproduction of soil microorganisms^40^ compared with GM. Similarly, CDB + GM and WB + GM increased the abundance of soil microbes compared to their sole forms due to similar reasons. Cao et al.^41^ also reported an increase in abundance of bacteria, fungi, and actinomycetes as a result of biochar and compost amendment applications.

The increased yield and growth parameters of tiger nut in this study as a result of the amendments were due to improved chemical characteristics of the soil as a result of biochar and GM applications. The increase in cations in biochar-amended plots brings an improvement in soil fertility and nutrient retention^42^ especially N and K that are important for tuber formation. Biochar could have reduced potassium leaching and promote the release of adsorbed soil potassium in the soil^43,44^.

Tiger nut performance with biochar application can be adduced to the optimization of the available plant nutrients^45,46^. Biochar increased the microbial and nutrient contents of the soil by changing soil physical properties^47^, thereby increasing growth and yield. The high microbial abundance may have led to high nutrient availability to crops through enhancing both the microbial biomass turnover and the degradation of non-microbial organic materials^48^.

CDB and CDB + GM increased growth and yield compared with WB and WB + GM. This can be related to the chemical composition (Table 2) and soil chemical properties (Table 3) of cow dung biochar. CDB has an enhanced CEC compared with WB. CEC shows the capacity of biochar to adsorb cation nutrients. Therefore, the addition of biochar (CDB) with higher CEC improves soil productivity by reducing nutrient leaching^33^ thereby making nutrients available to tiger nut hence improved growth and yield. Biochar produced from nutrient-rich feedstock such as animal manure has been reported to have higher nutrient content than biochar produced from lignin-rich plant biomass feedstock^49^. Alburquerque et al*.*^50^ also reported that nutrient-poor feedstock biochar may have limited soil fertility benefits in the short term leading to little improvement in crop growth. Uzoma, et al.^51^ reported an increase of 98–150% of maize yield due to manure biochar addition, also, Viger et al.^52^ recorded 111% of lettuce and Arabidopsis plant biomass increase as a result of poplar wood chips biochar addition.

GM improved growth and yield of tiger nut compared with the two biochar treatments. This could be related to the improved soil chemical properties of GM compared with CDM and WB. Improved yield and growth in GM treatment can also be related to its lowest C: N ratio, which would have enhanced faster nutrient release.

CDB + GM and WB + GM increased growth and yield of tiger nut compared with their sole forms. Biochar is known to be resistant to degradation and absorb nutrients, therefore the humus of biochar may allow retention of released nutrients from rapidly decomposing *Tithonia* with low C: N ratio (GM) within the rooting zone, thereby fostering greater efficiency of nutrient uptake and increase in yield. Therefore, biochar may store nutrients and then start to release them slowly like a slow-release fertilizer. Thus indicating a synergistic relationship between the inputs. Partey et al.^53^ reported that percent dry weight remaining after the first week of decomposition of *Tithonia* was 20% and the highest percent of nutrients (N, P, K, Ca, and Mg) in *Tithonia* are released during the first week of application. These release nutrients are not let go by the biochar but are “detained” for tiger nut use in addition to its own (biochar) nutrient. Greater synchronization of nutrient supply is considered one of the challenges facing organic resource management^54,55^ and the observed differences in decomposition rate between biochar and *Tithonia* leaves may present an opportunity in that regard.

Sole application of CDB, WB, and GM and combinations CDB + GM and WB + GM increased moisture, fiber, protein, and ash contents of tiger nut compared with the control. This might be as a result of an increase in the nutrients in the soil due to applications of these treatments which consequently led to increased absorption of nutrients by the plants. The CDB, WB, GM and their combinations applied would have increased N supply to the soil and consequently absorbed by the tiger nut plant and hence increased the number of leaves and photosynthetic activity and enhancing physiological processes leading to the production of more assimilates which leads to increase in the chemical composition of the tiger nut tubers. Moisture in the tiger nut plant increased because N in the amendments could have stimulated better growth and development of roots, good vegetative growth thereby stimulating greater absorption of water.

The protein content of tiger nut increased because N in the soil increased significantly as a result of this treatment relative to the control, N is known to be an integral component in plants including amino acids that are building blocks of protein and enzymes that are involved in catalyzing most biochemical processes^56^.

Lipids decreased with the amendment, this may be a consequence of diverting more energy and resources into protein production rather than oil^57^, leading to oil concentration accumulation reduction. It has also been reported^58^ that there is a negative correlation between oil and protein content.

CDB + GM improved moisture, ash, fiber, and protein more than other treatments because of better soil nutrient concentration which resulted in better nutrient absorption, growth, and better assimilates for quality tiger nut tuber.

## Conclusion

This study revealed that the application of CDB, WB, and GM either sole or combined increased moisture content, SOC, nutrient content, soil culturable microorganisms, growth, yield, moisture, ash, fiber, and protein contents of tiger nut compared with the control. Biochar from cow manure (CDB) has a higher N, C: N ratio, P, K, Ca, Mg, CEC, and pH contents when compared with wood biochar WB. CDB alone and CDB + GM increased growth and yield compared with WB alone and WB + GM which was adduced to the fact that CDB has an enhanced CEC compared with WB. GM alone improved growth and yield of tiger nut compared with the two sole biochar treatments. CDB + GM and WB + GM increased growth and yield of tiger nut compared with their sole forms. This was adduced to biochar allowing the retention of released nutrients from rapidly decomposing *Tithonia* with low C: N ratio (GM) within the rooting zone, thereby fostering greater efficiency of nutrient uptake and increase in yield. Therefore, for good soil fertility and tiger nut yield, it is important that the addition of a fast releasing nutrient source to biochar be sought.

## Materials and methods

### Site description and treatments

Two field experiments were conducted concurrently (site A and B) at the Teaching and Research Farm, Landmark University, Omu-Aran, Kwara State, Nigeria during the cropping season of 2019.

Experiment at site B was conducted simultaneously as A so as to validate the results of experiment A. Landmark University lies between lat 8° 9ʹN and long 5° 61ʹE at an altitude of 560 m and is located in the derived savanna ecological zone of Nigeria. The rainfall pattern is bimodal with peaks in June and October. The total annual rainfall in the area is about 1300 mm while the mean annual temperature is 32 °C. The soil at the site of the experiment is an Alfisol classified as Oxic Haplustalf or Luvisol^20^.

At both sites A and B, the treatments consisted of: (1) cow dung biochar applied alone at 10 t ha^−1^(CDB), (2) wood biochar applied alone at 10 t ha^−1^(WB), (3) Green manure—mexican sunflower (*Tithonia diversifolia* Asteraceae) applied alone at 10 t ha^−1^(GM), (4) cow dung biochar applied at 5 t ha^−1^ + green manure applied at 5 t ha^−1^(CDB + GM), (5) wood biochar applied at 5 t ha^−1^ + green manure applied at 5 t ha^−1^ (WB + GM), (6) control, no amendment whatsoever (C). The six treatments were arranged in a randomized complete block design with three replications. Each block comprised of 6 plots and each plot was 3 × 2 m. Blocks were 1 m apart and plots were 0.5 m apart.

### Incorporation of biochar and green manure and planting tiger nut tubers

The cow dung used as biochar was obtained from the animal section of the Teaching and Research Farm of Landmark University while the wood for the biochar was obtained from parkia (*Parkia biglobosa*) tree around the farm. They were both dried properly under natural conditions. After drying, the wood was cut into pieces. The cow dung and the wood were pyrolysed using a box-type resistance furnace through a slow pyrolysis at a temperature of 400 °C for 4 h and the biochar was then cooled for 12 h. The prepared biochar is then crushed and sieved through a 2 mm sieve.

Land preparation was by ploughing and harrowing, the site was then laid out to the required plot size of 3 × 2 m after which raised beds were constructed. The cow dung and wood biochar were weighed and spread evenly on the plots according to the required rates. A hand-held hoe was used to work the biochars into the soil to a depth of approximately 10 cm. The green manure (*Tithonia diversifolia*) used was harvested from nearby bushes in the Research Farm of the University with leaves and tender stems chopped and similarly incorporated using a hoe to the depth of 10 cm. The plots were left for 3 weeks before planting of tiger nuts to allow for decomposition of the amendments. The tiger nuts tubers to be planted were purchased from the market, tubers of uniform sizes were selected, and were tested for viability using floatation method, with the floating tubers discarded. Tubers were also soaked in water for 24 h before plating. Planting at both sites were done in May 2019, tubers were planted per hole at an inter-row spacing of 0.2 m and 0.6 m intra-row spacing to give a plant population of 50 plants per plot and 83,333 plants per ha. Weeding was done manually on a weekly basis.

### Determination of soil properties

At both sites before the start of the experiment, soil samples from topsoil (0–15 cm) were taken from random spots in the study area and were bulked together to form a composite sample. A viable cell count of fungi, actinomycete, and bacteria were carried out by spread plating samples onto on malt agar, actinomycete isolation agar media, and on standard plate count agar respectively^59^. Plates for fungi, actinomycete, and bacteria were thereafter incubated for 5 days, 7 days, and 1 day at 28 °C incubation temperatures. Five undisturbed samples (0.04 m diameter, 0.15 m high) were also collected at 0–0.15 m depth from five positions in each site at random using core steel sampler. The samples were used to evaluate bulk density after oven-drying at 100 °C for 24 h^20^. Collected and bulked soil samples were air-dried and sieved using a 2-mm sieve ready for analysis. The textural class of the soils was determined by the method of Gee and Or^60^. Soil organic carbon (OC) was determined by the procedure of Walkley and Black using the dichromate wet oxidation method^61^.

Total N was determined by the micro-Kjeldahl digestion method^62^. Available P was determined by Bray-1 extraction followed by molybdenum blue colorimetry^63^ Exchangeable K, Ca, and Mg was extracted using 1 M ammonium acetate^64^. K and Na in the extract were read on a flame photometer while Ca and Mg were read on Atomic Absorption Spectrophotometer. Soil pH was determined using a soil–water medium at a ratio of 1:2 with a digital electronic pH meter. At the termination of the experiment at both sites, soil samples were also collected on a plot basis. The CEC was determined by the BaCl_2_ compulsive exchange method as described by Gillman and Sumpter^65^ Soil moisture content was determined gravimetrically by oven drying at 105 °C overnight in addition to soil chemical properties and biological properties as described above.

### Analysis of biochar and green manure leaves

The CDB, WB, and GM used were analyzed for nutrient composition after the CDB and WB have being air-dried and crushed to pass through a 2-mm sieve. The analysis was done for organic carbon (OC) and total N, P, K, Ca, and Mg in accordance with AOAC^66^. CEC of the biochars were estimated using an NH4^+^ replacement method^67^. Leaf sample was collected from green manure, oven-dried for 24 h at 80 °C, and ground in a Willey mill. This sample was analyzed for N, P, K, Ca, and Mg as described by Tel and Hagarty^68^.

### Proximate analysis of tiger nut tubers

The moisture, ash, crude fiber, crude protein, lipid, and carbohydrate contents of the sweet potato were determined using standard chemical methods described by the Association of Analytical Chemists^66^. The ash content was determined by incineration of 2 g of each sample in a muffle furnace at 500 °C for 2 h. The moisture content was determined by drying 2 g of each sample at 105 °C till constant weight was achieved. Soxhlet extraction technique using petroleum ether (40–50 °C) was used to evaluate the lipid content of the samples. The crude protein content of the sample was determined by the micro-Kjeldahl digestion and distillation method^69^. The carbohydrate content of the sample was estimated by using the method described by Muller and Tobin^70^. The total carbohydrate was estimated as the balance after accounting for ash, crude fiber, protein, and fat. % carbohydrate = 100%—the sum of percentage moisture, ash, crude fat, crude fiber, and crude protein contents.

### Determinations of growth and yield parameters

Tiger nut growth (plant height and the number of leaves) was measured after 72 days after planting (flowering stage). Tiger nut plant height was measured using meter rule by measuring from the base of the plant in the soil to the last point on the leaf at the apex. Leaf numbers were counted. During harvesting—which is about 3 months after planting, each plant was uprooted using a shovel. Washing was done on a running tap water and then tubers were dried in open air after which they were counted and weighed.

### Statistical analysis

Data collected for soil chemical and biological properties, tiger nut’s yield, growth, and proximate compositions were subjected to one-way analysis of variance (ANOVA) using SPSS 17.0, and means were separated using Duncan’s multiple range test (DMRT) at *p* = 0.05 probability level.

## References

[1] Akoma O, Elekwa UO, Afodunrinbi AT, Onyeukwu G (2000). Yogurt from coconut and tiger nuts. J. Food Technol. Afr..

[2] Adejunitan JA (2011). Tiger nut processing: Its food, uses and health benefits. Am. J. Food Technol..

[3] Oladele AK, Aina JO (2007). Chemical composition and functional properties of flour produced from two varieties of tiger nut (*Cyperus ensculentus*). Afr. J. Biotech..

[4] Belewu MA, Abodunrin AO (2008). Preparation of kunun from unexploited rich food sources: tiger nut (*Cyperus esculentus*). Pak. J. Nutr..

[5] Defelice MS (2002). Yellow nutsedge (*Cyperus esculentus* L.) Snack food of the gods. Weed Technol..

[6] Oladele, A. K., Ibanga, U. I. & Adebesin, O. L. Effect of substituting maize with tigernut on the quality and acceptability of Dakuwa. In: *Proceedings of the 33rd Annual conference and General Meeting of Nigerian Institute of Food Science and Technology*, Yola, Nigeria. (2009).

[7] Salako FK, Hauser S, Babalola O, Tian G (2001). Improvement of the physical fertility of a degraded Alfisol with planted and natural fallows under humid tropical conditions. Soil Use Manag..

[8] Montgomery RF (1988). Some characteristics of moist Savanna soils and constraints on development with particular reference to Brazil and Nigeria. J. Biogeogr. Biogeogr. Dev. Humid Trop..

[9] Adekiya AO, Agbede TM, Ejue WS, Aboyeji CM, Dunsin O, Aremu CO, Owolabi AO, Ajiboye BO, Okunlola OF, Adesola OO (2020). Biochar, poultry manure and NPK fertilizer: sole and combine application effects on soil properties and ginger (*Zingiber officinale* Roscoe) performance in a tropical Alfisol. Open Agric..

[10] Ouyang L, Wang F, Tang J, Yu L, Zhang R (2013). Effects of biochar amendment on soil aggregates and hydraulic properties. J. Soil Sci. Plant Nutr..

[11] Amin FR, Huang Y, He YF, Zhang RH, Liu GQ, Chen C (2016). Biochar applications and modern techniques for characterization. Clean Technol. Environ. Policy.

[12] Adekiya, A.O., Agbede, T.M., Olayanju, A., Ejue, W.S., Adekanye, T.A., Adenusi, T.T. & Ayeni, J.F. Effect of biochar on soil properties, soil loss, and cocoyam yield on a tropical sandy loam Alfisol. *Sci. World J.* Volume 2020, Article ID 9391630, 9 pages. 10.1155/2020/9391630 (2020). 10.1155/2020/9391630 PMC7060867 32158364

[13] Glaser B, Lehmann J, Zech W (2002). Ameliorating physical and chemical properties of highly weathered soils in the tropics with charcoal—a review. Biol. Fertil. Soils.

[14] Liang B, Lehmann J, Solomon D (2006). Black carbon increases cation exchange capacity in soils. Soil Sci. Soc. Am. J..

[15] McClellan T, Deenik J, Uehara G, Antal M (2007). Effects of Flashed Carbonized Macadamia Nutshell Charcoal on Plant Growth and Soil Chemical Properties.

[16] Lehmann J, Joseph S, Lehmann J, Joseph S (2009). Biochar for environmental management: an introduction. Biochar for Environmental Management: Science and Technology.

[17] Liang B, Lehmann J, Solomon D (2009). Black carbon increases cation exchange capacity in soils. Soil Sci. Soc. Am. J..

[18] Cantrell KB, Hunt PG, Uchimiya M, Novak JM, Ro KS (2012). Impact of pyrolysis temperature and manure source on physicochemical characteristics of biochar. Bioresour. Technol..

[19] MacRae RJ, Mehuys GR (1988). The effect of green manuring on the physical properties of temperate-area soils. Adv. Soil Sci..

[20] Adekiya AO, Agbede TM, Aboyeji CM, Dunsin O, Simeon VT (2019). Effects of biochar and poultry manure on soil characteristics and the yield of radish. Sci. Hortic..

[21] Sonke D (1997). Tithonia weed—a potential green manure crop. Echo Dev. Notes.

[22] Adekiya AO (2019). Green manures and poultry feather effects on soil characteristics, growth, yield, and mineral contents of tomato. Sci. Hortic..

[23] Gachengo CN, Palm CA, Jama B, Othieno C (1999). Tithonia and Senna green manures and inorganic fertilizers as phosphorus sources for maize in western Kenya. Agrofor. Syst..

[24] Akinrinde, E. A. & Obigbesan, G. O. Evaluation of the fertility status of selected soils for crop production in five ecological zones of Nigeria. In: *Proceedings of the 26th Annual Conference of Soil Science Society of Nigeria*, pp. 279–288. (2000).

[25] Salako, F. K. Soil Physical Conditions in Nigerian Savannas and Biomass Production. Lecture given at the College on Soil Physics Trieste, 3–21 March 2003, 14 pp (2003).

[26] Adekiya AO, Ojeniyi SO (2002). Evaluation of tomato growth and soil properties under methods of seedling bed preparation in an Alfisol in the rainforest zone of southwest Nigeria. Soil Tillage Res..

[27] Thies JE, Rillig MC, Lehmann J, Joseph S (2009). Characteristics of biochar: biological properties. Biochar for Environmental Management.

[28] Glaser B, Haumaier L, Guggenberger G, Zech W (2001). The ‘Terra Preta’ phenomenon: a model for sustainable agriculture in the humid tropics. Naturwissenschaften.

[29] Lehmann, J., Da Silva Jr., J. P., Rondon, M., Da Silva, C. M., Greenwood, J., Nehls, T., Steiner, C. & Glaser, B. Slash-and-char: a feasible alternative for soil fertility management in the Central Amazon? In *Proceedings of the 17th World Congress of Soil Science, Symposium No*. 13, Paper No. 449 1–12 (2002).

[30] Mizuta K, Matsumoto T, Hatate Y, Nishihara K, Nakanishi T (2004). Removal of nitrate-nitrogen from drinking water using bamboo powder charcoal. Bioresour. Technol..

[31] Beaton JD, Peterson HB, Buer N (1960). Some aspect of phosphate adsorption by charcoal. Soil Sci. Soc. Am. J..

[32] Radovic LR, Moreno-Castilla C, Rivera-Utrilla J, Radovic LR (2001). Carbon materials as adsorbents in aqueous solutions. Chemistry and Physics of Carbon.

[33] Sohi S, Krull E, Lopez-Capel E, Bol R (2010). A review of biochar and its use and function in soil. Adv Agron..

[34] De Luca TH, MacKenzie MD, Gundale MJ, Lehmann J, Joseph S (2009). Biochar effects on soil nutrient transformations. Biochar for Environmental Management:Science and Technology.

[35] Major J, Steiner C, Downie A, Lehmann J, Lehmann J, Joseph S (2009). Biochar effects on nutrient leaching. Biochar for Environmental Management: Science and Technology.

[36] Rajkovich S, Enders A, Hanley K, Hyland C, Zimmerman AR, Lehmann J (2012). Corn growth and nitrogen nutrition after additions of biochars with varying properties to a temperate soil. Biol. Fertil. Soils.

[37] Shokalu AO, Ojo AO, Ezekiel-Adewoyin DT, Akintoye HA, Azeez JO (2010). Evaluation of *Tithonia diversifolia* for soil improvement in Celosia (*Celosia argentea*) production. Electron. J. Environ. Agric. Food Chem..

[38] Chan KY, van Zwieten L, Meszaros I, Downie A, Joseph S (2008). Using poultry litter biochars as soil amendments. Aust. J. Soil Res..

[39] Van Zwieten L, Kimber S, Morris S, Chan KY, Downie A, Rust J, Joseph S, Cowie A (2010). Effect of biochar from slow pyrolysis of papermill waste on agronomic performance and soil fertility. Plant Soil.

[40] Warnock DD, Mummey DL, McBride B, Major J, Lehmann J, Rillig MC (2010). Influences of non-herbaceous biochar on arbuscular mycorrhizal fungal abundances in roots and soils: results from growth-chamber and field experiments. Appl. Soil Ecol..

[41] Cao Y, Ma Y, Guo DJ, Wang QJ, Wang GF (2017). Chemical properties and microbial responses to biochar and compost amendments in the soil under continuous watermelon cropping. Plant Soil Environ..

[42] Cheng CH, Lehmann J, Thies JE, Burton SD, Engelhard MH (2006). Oxidation of black carbon by biotic and abiotic processes. Org. Geochem..

[43] Abujabhah IS, Doyle R, Bound SA, Bowman JP (2016). The effect of biochar loading rates on soil fertility, soil biomass, potential nitrification, and soil community metabolic profiles in three different soils. J. Soils Sediments.

[44] Pandian K, Subramaniayan P, Gnasekaran P, Chitraputhirapillai S (2016). Effect of biochar amendment on soil physical, chemical and biological properties and groundnut yield in rainfed Alfisol of semi-arid tropics. Arch. Agron. Soil Sci..

[45] Abiven S, Hund A, Martinsen V, Cornelissen C (2015). Biochar amendment increase maize root surface area and branches: a shovelonic study in Zambia. Plant Soil.

[46] Lehmann J, da Silva JP, Steiner C, Nehls T, Zech W, Glaser B (2003). Nutrient availability and leaching in an archaeological Anthrosol and a Ferralsol of the Central Amazon basin: fertilizer, manure and charcoal amendments. Plant Soil.

[47] Ding Y, Liu Y, Liu S, Li Z, Tan X (2016). Biochar to improve soil fertility: a review. Agron. Sustain. Dev..

[48] Wang Q, Wang Y, Wang S, He T, Liu L (2014). Fresh carbon and nitrogen inputs alter organic carbon mineralization and microbial community in forest deep soil layers. Soil Biol. Biochem..

[49] Filiberto DM, Gaunt JL (2013). Practicality of biochar additions to enhance soil and crop productivity. Agriculture.

[50] Alburquerque JA, Calero JM, Barrón V, Torrent J, del Campillo MC, Gallardo A, Villar R (2014). Effects of biochars produced from different feedstocks on soil properties and sunflower growth. J. Plant Nutr. Soil Sci..

[51] Uzoma KC, Inoue M, Andry H, Fujimaki H, Zahoor A, Ni shihara E (2011). Effect of cow manure biochar on maize productivity under sandy soil condition. Soil Use Manag..

[52] Viger M, Hancok RD, Milietta F, Taylor G (2015). More plant growth but less plant defence? First global gene expression data for plants grown in soil amended with biochar. GCB Bioenergy.

[53] Partey ST, Quashie-Sam SJ, Thevathasan NV, Gordon AM (2011). Decomposition and nutrient release patterns of the leaf biomass of the wild sunflower (*Tithonia diversifolia*): a comparative study with four leguminous agroforestry species. Agrofor. Syst..

[54] Myers RJK, Palm CA, Cuevas E, Guanatilleke IUN, Brossard M, Woomer PL, Swift MJ (1994). The synchronization of nutrient mineralisation and plant nutrient demand. The Biological Management of Tropical Soil Fertility.

[55] Palm, C. A., Myers, R. J. K. & Nandwa, S. M. Combined use of organic and inorganic nutrient sources for soil fertility maintenance and replenishment. In: Buresh, R. J., Sanchez, P.A.& Calhoun, F. (Eds.), *Replenishing Soil Fertility in Africa*. SSSA Special Publication Number 51, pp. 193–217 (1997).

[56] Brady NC, Weil RR (2008). The Nature and Properties of Soils.

[57] Solis GP, Radon Y, Sempou E, Jechow K, Stuermer CAO, Málaga-Trillo E (2013). Conserved roles of the prion protein domains on subcellular localization and cell-cell adhesion. PLoS ONE.

[58] Blumenthal J, Battenspenrger D, Cassman KG, Mason KG, Pavlista A, Hatfield JL, Folett RF (2008). Importance of nitrogen on crop quality and health. Nitrogen in the Environment: Sources Problems and Management.

[59] Alfano G, Lustrato G, Lima G, Vitullo D, Ranalli G (2011). Characterization of composted olive mill wastes to predict potential plant disease suppressiveness. Biol. Control.

[60] Gee, G. W. & Or, D. Particle-size analysis. In: Dane, J H. & Topp, G. C. (Eds.), *Methods of Soil Analysis. Part 4. Physical Methods*. Soil Science Society of America Book Series No. 5, Madison, WI, USA, pp. 255–293 (2002).

[61] Nelson, D. W. & Sommers, L. E. Total carbon, organic carbon and organic matter. In: Sparks, D. L. (Ed.), *Methods of Soil Analysis. Part 3*, 2nd edn. ASA and SSSA, Madison (WI), pp. 961–1010 SSSA Book Series No. 5 (1996).

[62] Bremner, J. M. Nitrogen-total. In: Sparks, D. L. (Eds.), *Methods of Soil Analysis: Part 3: Chemical methods*, 2nd edition. Soil Science Society of America, Book Series No. 5. ASA and Soil Science Society of America, Madison, Wisconsin, USA, pp. 1085‒1121 (1996).

[63] Frank, K., Beegle, D. & Denning, J. Phosphorus. In: Brown, J. R. (Ed.), *Recommended Chemical Soil Test Procedures for the North Central Region*. North Central Regional Research Publication No. 221. Missouri Agric. Exp. Station, Columbia (MO), pp. 21–26 (Revised) (1998).

[64] Hendershot, W. H., Lalande, H. & Duquette, M. Ion exchange and exchangeable cations. In: Carter, M. R. & Gregorich, E. G. (Eds.), *Soil sampling and methods of analysis*. Canadian Society of Soil Science, 2nd ed. CRC Press, Boca Raton (Florida), pp. 197–206 Chapter 18 (2007).

[65] Gillman GP, Sumpter EA (1986). Modification to the compulsive exchange method for measuring exchange characteristics of soils. Soil Res..

[66] AOAC, Official Methods of Analysis. Association of Official Agricultural Chemists International, Washington, D.C. (2010).

[67] Schollenberger CJ, Simon RH (1945). Determination of exchange capacity and exchangable bases in soil-ammonium acetate method. Soil Sci..

[68] Tel, D. A. & Hagarty, M. Soil and Plant Analysis. Study Guide for Agricultural Laboratory Directors and Technologists Working in Tropical Regions. International Institute of Tropical Agriculture (IITA), Ibadan, Nigeria in conjunction with University of Guelph, Ontario, Canada 277 pp. (1984).

[69] Tomov, T., Rachovski, G., Kostadinova, S. & Manolov, I. Handbook of Agrochemistry. Columbia, MO 2009, 109 (2009).

[70] Muller, H. G. & Tobin, G. Nutritional and food processing. Taylor and Francis Inc., London, UK, ISBN-13:9780870553639, pp 152 (1980).

